# Structure and decay of a proto-Y region in Tilapia, *Oreochromis niloticus*

**DOI:** 10.1186/1471-2164-15-975

**Published:** 2014-11-17

**Authors:** William J Gammerdinger, Matthew A Conte, Enoch A Acquah, Reade B Roberts, Thomas D Kocher

**Affiliations:** Department of Biology, University of Maryland, College Park, MD 20742 USA; Program in Genetics, North Carolina State University, Raleigh, NC 27695 USA

## Abstract

**Background:**

Sex-determination genes drive the evolution of adjacent chromosomal regions. Sexually antagonistic selection favors the accumulation of inversions that reduce recombination in regions adjacent to the sex-determination gene. Once established, the clonal inheritance of sex-linked inversions leads to the accumulation of deleterious alleles, repetitive elements and a gradual decay of sex-linked genes. This in turn creates selective pressures for the evolution of mechanisms that compensate for the unequal dosage of gene expression. Here we use whole genome sequencing to characterize the structure of a young sex chromosome and quantify sex-specific gene expression in the developing gonad.

**Results:**

We found an 8.8 Mb block of strong differentiation between males and females that corresponds to the location of a previously mapped sex-determiner on linkage group 1 of *Oreochromis niloticus*. Putatively disruptive mutations are found in many of the genes within this region. We also found a significant female-bias in the expression of genes within the block of differentiation compared to those outside the block of differentiation. Eight candidate sex-determination genes were identified within this region.

**Conclusions:**

This study demonstrates a block of differentiation on linkage group 1, suggestive of an 8.8 Mb inversion encompassing the sex-determining locus. The enrichment of female-biased gene expression inside the proposed inversion suggests incomplete dosage compensation. This study helps establish a model for studying the early-to-intermediate stages of sex chromosome evolution.

**Electronic supplementary material:**

The online version of this article (doi:10.1186/1471-2164-15-975) contains supplementary material, which is available to authorized users.

## Background

The classic model of sex chromosome evolution begins with the emergence of a new sex- determining gene on an autosome [1]. The new sex-determiner may be linked with genes experiencing sexually antagonistic selection. Selection favors mechanisms, such as chromosomal inversions, that reduce recombination between the sex-determination locus and sexually antagonistic genes [2, 3]. The human sex chromosomes have undergone at least four such inversions, which may have limited recombination between the sex-determination locus and nearby sexually antagonistic genes [4].

Inversions create a clonally inherited chromosomal segment with a relatively small effective population size when compared to the rest of the genome [5]. Sex chromosomes therefore become a haven for deleterious mutations and repetitive elements that are difficult to purge. These deleterious mutations accumulate via Muller’s Ratchet, as well as by hitchhiking with advantageous mutations [6, 7]. Degradation of functional genes on the Y- or W-chromosome leaves the homogametic sex carrying two functional copies of a particular gene, while the heterogametic sex carries only one functional copy. Therefore, mechanisms are needed to maintain appropriate expression of dosage-sensitive genes on emerging sex chromosomes [8–10]. In mammals, global dosage compensation is accomplished through X-inactivation [10, 11]. However, in many species, dosage compensation is partial and the expression of many genes is not compensated [8].

Some sex-determining genes are conserved for long periods of time. An example is *Sry*, a gene that has controlled sex-determination in therian mammals for approximately 180 million years [7, 12–14]. Other sex-determination genes hold sway for much shorter periods of time. There have been at least five transitions in the mechanism controlling sex-determination in rice fish (genus *Oryzias*) during the last 20 million years [15]. Similarly rapid rates of sex chromosome evolution have been identified among sticklebacks (Family Gasterosteidae) [16].

The evolution of new sex-determination genes may have contributed to the rapid radiation of African cichlid fishes [17]. Among the closely related haplochromine cichlids of Lake Malawi, sex-determination regions have been localized to linkage groups 3 (ZW), 5 (ZW), 7 (XY) and 20 (XY) [18–20]. Among tilapia cichlids, sex-determination regions have been localized to linkage groups 1 (XY), 3 (ZW) and 23 (XY) [21, 22]. Multiple sex-determination genes often segregate within a single species [18, 23]. The blue tilapia, *Oreochromis aureus*, segregates both an XY system on linkage group 1 and a ZW system on linkage group 3 [23]. Some strains of the Nile tilapia, *O. niloticus*, have an XY system on linkage group 1, while others segregate an XY system on linkage group 23 [22, 24, 25].

The goal of this study was to characterize the sex-determination locus on linkage group 1 in *O. niloticus*. We took a family-based strategy, separately pooling males and females from two crosses, and performing whole genome sequencing on the pooled DNAs. We cataloged the density and frequency of single nucleotide polymorphisms (SNPs) and assessed their functional impact. We identified an 8.8 Mb block of differentiation suggestive of a Y-linked inversion on linkage group 1. We found high densities of functionally significant SNPs within this differentiated block. Analysis of gonadal transcriptomes demonstrated an enrichment of female-biased gene models within the inversion, which suggests that dosage compensation is incomplete in this strain of *O. niloticus*.

## Results and discussion

### Sequencing of male and female DNA pools

We obtained ~202 million reads from the pool of male DNA and ~219 million reads from the pool of female DNA. 90.12% of the male and 90.67% of the female reads were aligned to the *O. niloticus* reference genome. Genome-wide coverage was slightly lower in males (32.97, standard deviation = 24.41 alignments per site), compared to females (36.68, standard deviation = 31.39 alignments per site).

### Large block of divergence on linkage group 1

**Figure 1:**
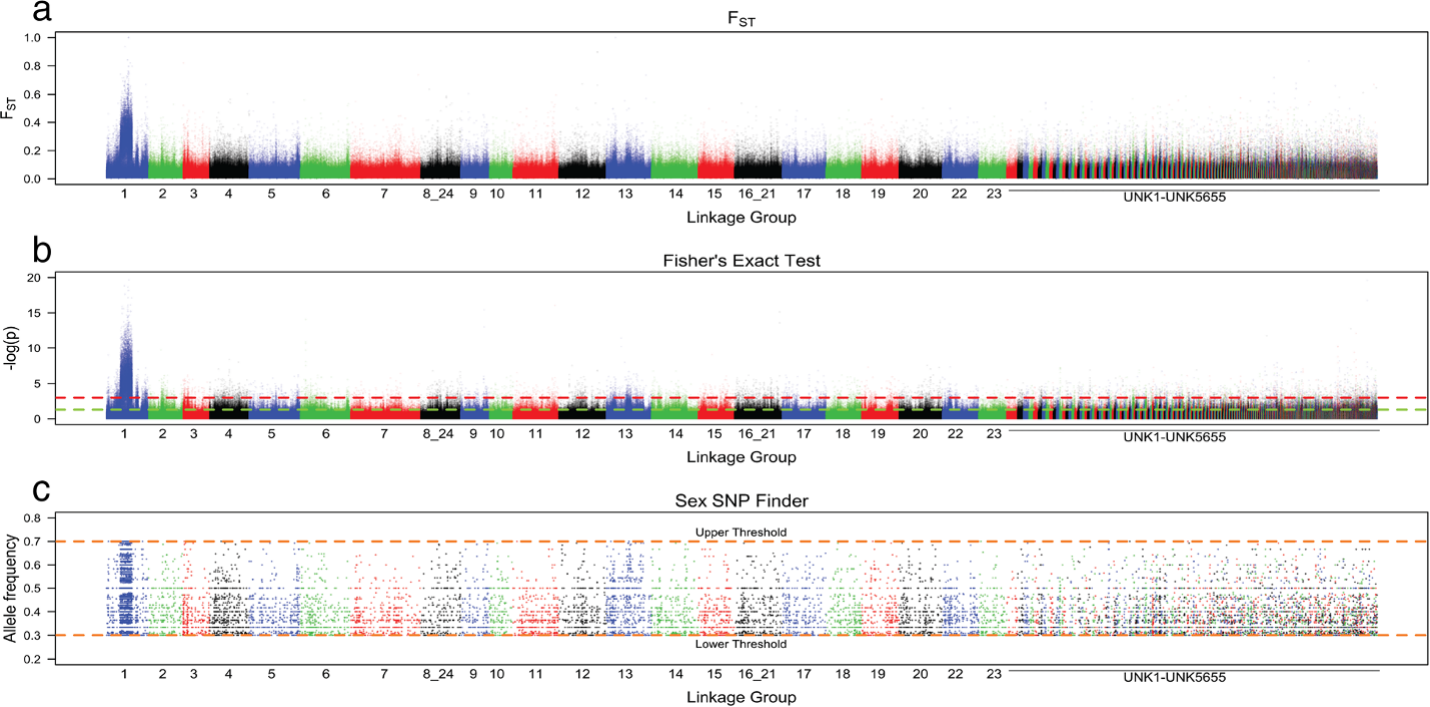
**Genome-wide scan for population differentiation.** Genome-wide statistics for **(a)** F_ST_, **(b)** Fisher’s Exact Test and **(c)** intermediate frequency SNPs in males that are fixed or nearly fixed in females.

**Figure 2:**
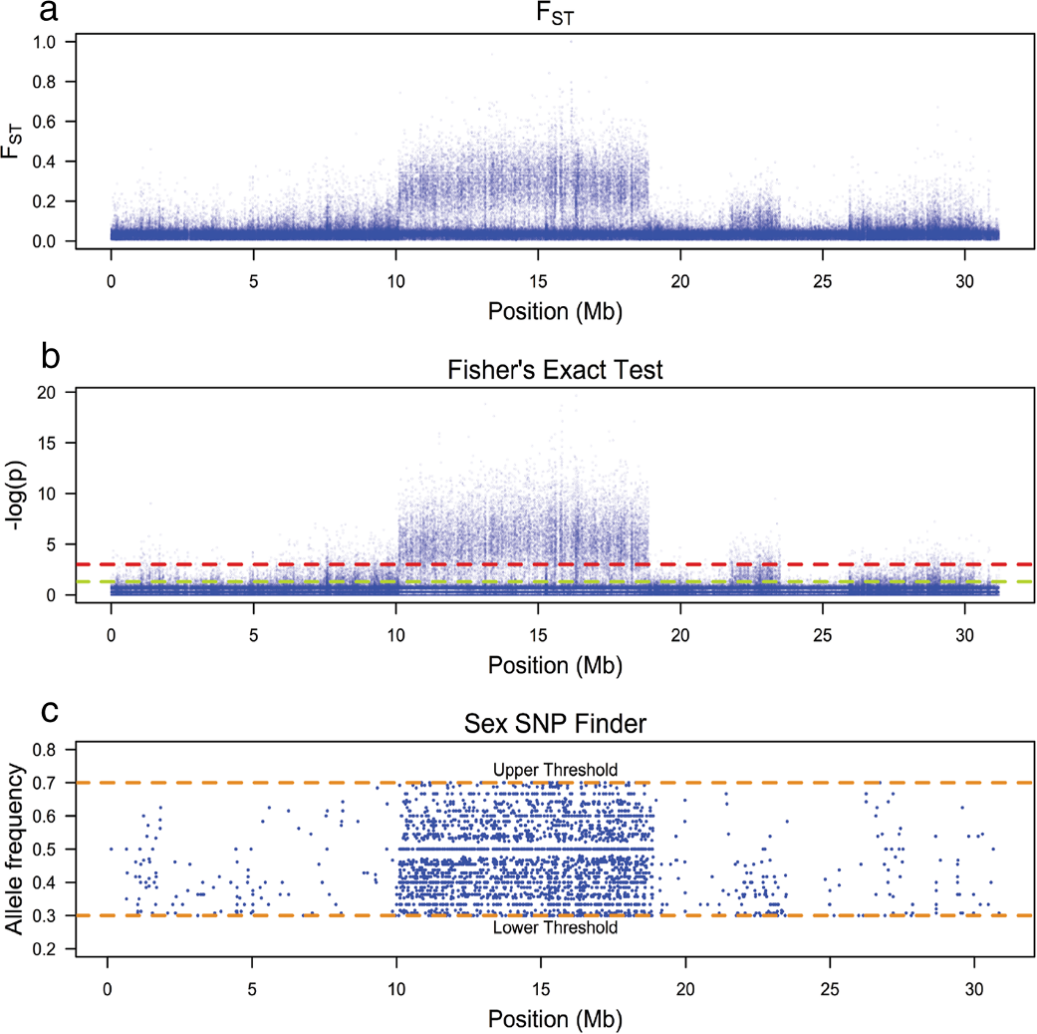
**Population differentiation on linkage group 1.** Differentiation statistics for linkage group 1. **(a)** F_ST_, **(b)** Fisher’s Exact Test and **(c)** intermediate frequency SNPs in males that are fixed or nearly fixed in females.

The mean F_ST_ between the male- and female-pooled genomes at polymorphic sites over the entire genome was 0.0356 (standard deviation = 0.030). A region between 10.1 Mb and 18.9 Mb on linkage group 1 showed a substantially higher value of F_ST_ = 0.0807 (standard deviation = 0.061) (Figures 1a, and 2a). This region corresponds to the previously mapped sex-determination region in this strain of *O. niloticus* [21, 24–27]. Mean read coverage within the differentiated region was lower in males (34.65, standard deviation = 10.56), compared to females (38.45, standard deviation = 12.00), but this difference was consistent with the total number of reads obtained from each sex. We used Fisher’s exact test to determine whether the allele frequency of SNPs was significantly different between males and females. We found a cluster of highly significant SNPs within the differentiated block on linkage group 1 (Figures 1b, and 2b).

We also counted the number of positions per 10 kb window that were fixed in female pools and had a SNP in intermediate frequency in male pools, as would be consistent with females having two X chromosomes and males having an X and a Y chromosome, using Sex_SNP_finder_now.pl. There were 40,514 of these SNPs found across the genome. 18,277 (2,076.932/Mb) lay inside the differentiated block and 22,237 (24.197/Mb) lay outside. Among the 300 non-overlapping 10 kb windows with the highest frequency of these SNPs, 290 were found within the differentiated block on linkage group 1. The mean number of such SNPs per window was 21.81 (standard deviation = 13.84) within the differentiated block and only 0.33 (standard deviation = 1.29) outside of this region (Figures 1c, and 2c). The elevated F_ST_, along with the abundance of intermediate frequency SNPs in males that are fixed in females, suggests that this region has limited, if any, recombination between the X and Y alleles.

We considered the possibility that this block of differentiation is an artifact of the process by which we selected individuals for sequencing. We initially screened individuals by genotyping two sex-linked microsatellites in order to confirm family identity and sex. We required males to demonstrate a heterogametic pattern and females to demonstrate a homogametic pattern for both markers. Five male and five female individuals were excluded by these criteria and may represent naturally sex-reversed individuals. The sharply defined edges of the block lie 4.22 Mb upstream and 3.37 Mb downstream of the microsatellites we genotyped (Figures 1 and 2), which would normally represent approximately 5 cM of genetic distance in this species [28]. However, there is no evidence of an exponential decay of F_ST_ in the flanking regions as would be expected if there was recombination between the markers and the edges of the block. We also considered the possibility that the high level of differentiation might be due to an 8.8 Mb duplication on the Y. However, the depth of read coverage is relatively consistent across this entire linkage group. Additionally, cytogenetic studies have not revealed any evidence of heteromorphy in this chromosome pair as would arise from a translocation [29]. The sum of the evidence suggests that this block of differentiation most likely reflects an 8.8 Mb inversion on the Y-chromosome.

The relatively small size of the putative inversion, and its location in the middle of the chromosome, make it challenging to characterize using standard cytogenetic techniques. Ideally, we would characterize the breakpoints, but we were unable to identify anomalous Illumina mate pairs near the ends of the inversion in our short insert libraries. Longer reads or more widely spaced mate pairs will be needed to characterize the breakpoints of the proposed inversion.

### Functionally significant SNPs

We examined the functional consequences of the SNPs that were fixed in female pools but at intermediate frequency in male pools at the same position using SnpEff and SnpSift [30, 31]. Within the 8.8 Mb differentiated block we found 13 stop codon changes (1.477/Mb), 3 start codon losses (0.341/Mb) and 2 splice site alterations (0.227/Mb, Table 1). In the remaining 919 Mb of the genome we found a total of 9 stop codon changes (0.010/Mb), no start codon losses, and 3 splice site alterations (0.003/Mb, Additional file 1). SNPs classified as non-synonymous coding changes by SnpEff totaled 168 (19.091/Mb) within the differentiated region and 147 (0.160/Mb) across the rest of the genome (Additional file 2).

**Table 1:** **Putative functional mutations in the proposed inversion**

Gene name	SNP location on LG1	Reference codon	SNP codon	Effect on the Y	Effect on the X	Frequency of SNP in males	Frequency of SNP in females
Ras-related protein R-Ras2 (LOC100693950)	10506882	CGA	**T**GA	Stop Gain	-	0.462	0
Signal peptide	10868192	TCA	T**G**A	-	Stop Gain	0.545	1
AMP deaminase 3 (LOC100694225)	11096201	TGA	T**C**A	Stop Lost	-	0.454	0
Zinc finger protein 821 (LOC100712266)	12466312	ATG	A**C**G	Start Lost	-	0.4	0
Zinc finger protein 821 (LOC100712266)	12466313	ATG	AT**A**	Start Lost	-	0.4	0
SAFB-like transcription modulator (LOC100711186)	12619332	CGA	**T**GA	-	Stop Gain	0.613	1
Hepatic lipase (Lipc)	12690753	TGA	**C**GA	Stop Lost	-	0.448	0
Ammonium transporter Rh type C 2 (LOC100706367)	13529856	ATG	AT**A**	-	Start Lost	0.575	1
AFG3-like protein 1 (LOC100702885)	13725056	TTA	T**G**A	Stop Gain	-	0.439	0
CUB and sushi domain-containing protein 1 (LOC100698036)	15189214	CAA	**T**AA	Stop Gain	-	0.391	0.027
CUB and sushi domain-containing protein 1 (LOC100698036)	15243948	TGC	TG**A**	Stop Gain	-	0.391	0
Neuromedin-K receptor (LOC100693904)	15788009	CAGG	CAG**C**	Splice Site Acceptor Lost	-	0.343	0
Protein FAM176A (LOC100700039)	16417062	CGA	**T**GA	Stop Gain	-	0.475	0
GC-rich sequence DNA-binding factor (LOC100700589)	16480688	CAGA	CAG**G**	Splice Site Acceptor Gain	-	0.512	0.018
BTB/POZ domain-containing protein KCTD3 (LOC100703295)	16809270	CGA	**T**GA	Stop Gain	-	0.52	0
Hypothetical protein (LOC100705710)	17489648	CAA	**T**AA	-	Stop Gain	0.452	0.97
Hypothetical protein (LOC100705710)	17507222	TAA	**C**AA	Stop Lost	-	0.469	0.04
Nuclear factor of activated T-cells	18194412	TGG	TG**A**	-	Stop Gain	0.5	0.982

Genes containing a stop codon, start codon or splice site alterations that were in intermediate frequency in males and fixed or nearly fixed in females within the proposed inversion on LG1. Bold denotes the altered SNP.

The elevated density of high impact SNPs within the proposed inversion leads us to believe that deleterious alleles have begun to accumulate on this proto-Y. This is in accordance with the canonical model of heterogametic sex-chromosome evolution [2, 32] and empirical observations of the therian mammal Y-chromosome, *Silene*, *Drosophila* and tongue sole [4, 33–35].

### Localization of the sex-determining gene

**Figure 3:**
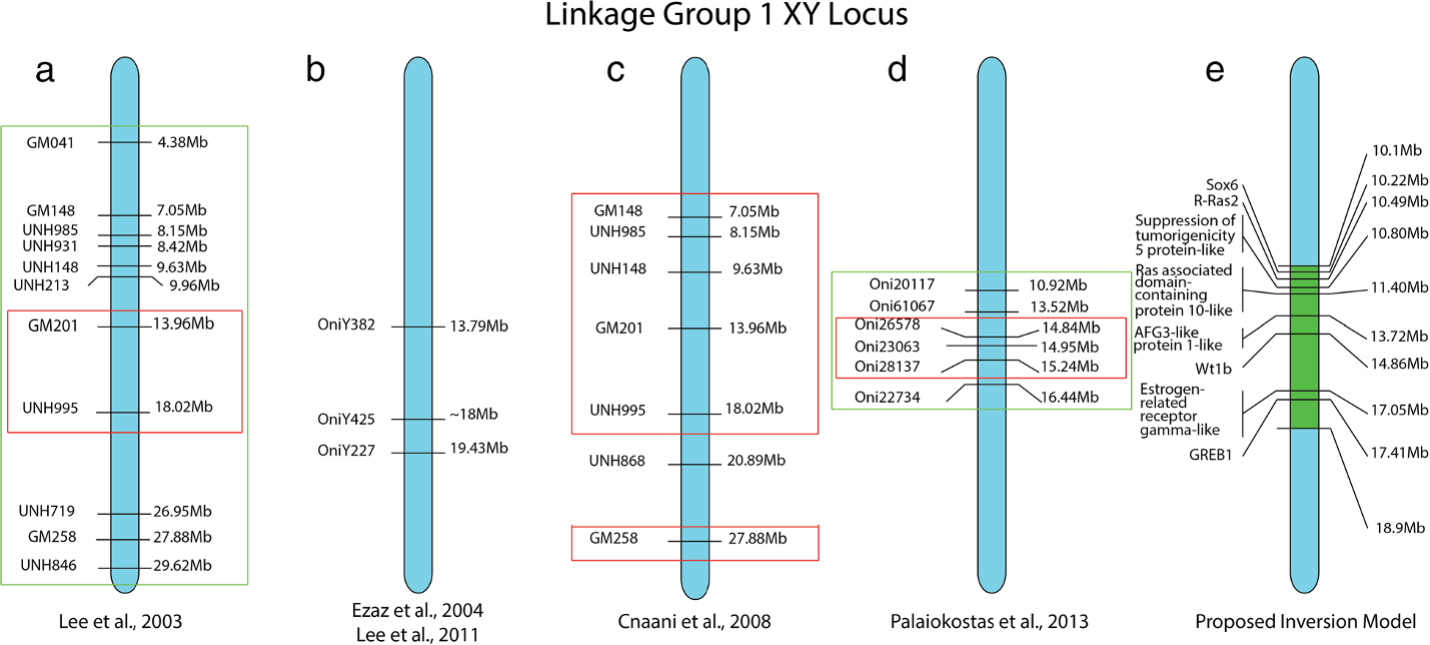
**Mapping of sex-determination locus on linkage group 1.** Previous studies identifying sex-linked markers on LG1. **(a)** Lee et al., [24] used a bulked segregant analysis. The green rectangle surrounds markers that were significantly sex-associated. The red rectangle encompasses the region with the highest significance. **(b)** Ezaz et al., [26] identified three Y-specific AFLPs. OniY425 was assigned through BLAST to scaffold UNK43. It was placed on LG1 according to Lee et al., [25], which used BAC contigs to place it within 100 kb of UNH995. **(c)** Cnaani et al., [21], also used a bulked segregant analysis. The markers within the red rectangles indicate markers that were significantly associated with sex. **(d)** Palaiokostas et al., 2013, identified sex-linked RAD-Seq markers. The green rectangle encompasses the markers with a LOD score greater than 15, while the red rectangle encloses the markers flanking the marker with the highest LOD score (Oni23063 with a LOD score of 18.5). **(e)** Proposed inversion in green with the eight candidate genes discussed in this paper.

Previous studies have concluded that sex is multifactorial in *O. niloticus* [24, 36] with a major sex-determination gene on LG1 [21]. Our study confirms this previous work, identifying an XY sex-determination locus in the middle of LG1 (Figure 3). The sex-determination gene was first mapped near microsatellite markers GM201 (13.96 Mb) and UNH995 (18.02 Mb, Figure 3a) [24]. Additional AFLP and FISH mapping found sex-associated markers at 13.79 Mb, near 18 Mb and at 19.43 Mb (Figure 3b) [25, 26]. Another study confirmed GM201 and UNH995 along with several other sex-associated markers spanning a region from 7.05 Mb to 18.02 Mb (Figure 3c) [21]. Lastly, a RAD-seq experiment found the highest associations at 14.95 Mb (LOD score 18.5), but demonstrated a broad region spanning 10.92 Mb to 16.44 Mb with a LOD score above 15 (Figure 3d) [27].

The multifactorial nature of sex-determination in this species causes difficulties for genetic mapping studies. An XX individual may develop as a male due to other genetic factors, or environmental effects on differentiation. These individuals would appear to be recombinant in the sex interval. We previously claimed to exclude *Wilm’s tumor protein homolog* (*Wt1b*) as the sex-determining gene on the basis of two recombinant individuals [37], but this conclusion is now in doubt. Conversely, the absence of recombination within the proposed inversion may preclude any further fine-mapping of the gene responsible for sex-determination.

### Differences in gene expression

The block of differentiation on linkage group 1 comprises just more than 1% of the assembled genome and contains 257 RefSeq annotated genes. Cufflinks predicted 234 gene models within the block of differentiation and predicted 22,411 gene models across the entire transcriptome. Of the gene models that showed an FPKM of >0.05 in at least one sex, 7,977 gene models (37.4%) showed higher expression in males, while 13,375 (59.7%) gene models showed higher expression in females. Furthermore, within the inverted region, only 68 of these gene models (29.6%) showed a male bias (Additional file 3), while 162 of these gene models (69.2%) showed a female bias (Additional file 4). The enrichment of female biased gene models within the proposed inversion is significant (χ^2^ = 5.58, p <0.05). These data suggest that this sex chromosome is at an early-to-intermediate evolutionary phase where the degradation of a proto-Y has begun and expression of Y-linked genes in males is reduced. However, mechanisms for complete dosage compensation have yet to take hold.

### Candidate sex determiners

Since the proposed inversion limits further attempts to fine-map the sex-determination gene, we evaluated candidate genes based upon putative functional polymorphisms, differential expression and prominence in pathways considered critical to sex-determination in other species. The complete list of candidate genes is presented in Table 2.

**Table 2:** **Candidate genes in the proposed inversion**

Gene name	Gene location on LG1 (Mb)	SNP location on LG1	Coding change	Pool frequency in males	Pool frequency in females	Male FPKM	Female FPKM
Transcription factor SOX-6 (LOC100694759)	10.22-10.30	10295869	T789K	0.3684	1	3.56951	4.24045
Ras-related protein R-Ras2 (LOC100693950)	10.48-10.51	10506882	R94STOP	0.4615	0	6.78248	5.16947
Suppression of tumorigenicity 5 protein (LOC100693420)	10.80-10.85	-	-	-	-	7.41571	2.09969
Ras association domain-containing protein 10 (LOC100693148)	11.40-11.41	-	-	-	-	0.252165	0.0688204
AFG3-like protein 1 (LOC100702885)	13.72-13.73	-	-	-	-	3.35696	0.36143
Wilms tumor protein homolog (LOC100701078)	14.86-14.88	14873730	A237V	0.4545	0	22.5644	13.2172
Estrogen-related receptor gamma (LOC100704106)	17.05-17.11	17093619	R172H	0.4333	0.06	0.370072	0.105597
Growth regulation by estrogen in breast cancer 1 (GREB1)	17.41-17.42	17424470	R1775C	0.5333	0.0571	0.961782	0.65826

Sex-determination candidate genes within the proposed inversion with any codon changes and their FPKM values.

First, we analyzed all SNPs that SnpEff classified as high impact mutations (Table 1). One prominent candidate within the proposed inversion is *Ras-related protein R-Ras2* (10.49 Mb-10.51 Mb), which is part of the Ras-MEK-ERK pathway within the TGF-β signaling network [38]. Alterations to the TGF-β network have been suggested as the mechanism for sex-determination in several fish species [15]. *Ras2* has been implicated as particularly important in the proliferation of cells [39] and is expressed during early development in a hermaphroditic fish, *Kryptolebias marmoratus* [40]. *Ras-related protein R-ras2* has a stop codon gain in intermediate frequency in males that is absent in females. Disruption of *R-ras2* could lead to decreased cell proliferation of primordial germ cells, resulting in increased likelihood of maleness [15, 39].

Next, we evaluated SNPs that SnpEff categorized as missense mutations (Additional file 2). The first of these candidate genes is *Wilms tumor protein homolog*, *Wt1b* (14.86 Mb-14.88 Mb), which has been implicated in gonadal development and acts directly upstream of *AMH*, the sex-determination gene in *Odontesthes hatcheri* [41]. *Wt1b* has also been demonstrated to bind to DNA and upregulate the sex-determination gene *Sry* in mammals. There is an A237V missense mutation in *Wt1b* that is absent in females and in intermediate frequency in males. Although our previous paper rejected *Wt1b* on the basis of two recombinant individuals [37], in light of the proposed inversion, we now believe that these individuals represented instances of natural sex reversal, not recombination.

A third candidate gene is *estrogen-related receptor gamma*, *ERR*γ (17.05-17.11 Mb). It has a R172H missense mutation within a predicted DNA-binding domain [42]. *ERR*γ has been shown to be a transcriptional activator of *DAX-1*, and *DAX-1* has been implicated as having an antagonistic effect to *Sry* in mammals [43]. Therefore, a mutation in the DNA-binding domain of *ERR*γ could reduce *DAX-1* transcription and thus have a masculinizing effect.

*Growth regulation by estrogen in breast cancer 1* (*GREB1*) is another candidate gene (17.41-17.42 Mb) with a missense mutation. The R1775C mutation alters the side chain from a basic side chain to a polar side chain. *GREB1* has been shown to be predominantly expressed within ovaries of young mice [44]. Additionally, *GREB1* has been demonstrated to be a coactivator of estrogen receptor-α [45]. Therefore, the missense mutation in *GREB1* could downregulate the expression of estrogen receptor-α, resulting in a masculinizing effect on the developing embryo.

Another potential sex-determination gene is *transcription factor SOX-6* (10.22 Mb-10.30 Mb). There is a T789K missense mutation in intermediate frequency in males that is fixed in females and changes a polar side chain into a basic one. *SOX-6* protein is localized to the same nuclear speckles as *Sry* and it has been suggested that it might play a role in sex-specific splicing in mammals [46].

We also evaluated gene models showing differential expression between males and females (Additional files 3 and 4). *AFG3(ATPase Family Gene 3)-like protein 1* (13.72 Mb-13.73 Mb) has over a nine-fold male-biased expression. It is also on the list of SNPs with high impact coding alterations with a stop codon gain. However, a clear tie to sex-determination has yet to be elucidated.

*Suppression of tumorigenicity 5 protein* (10.80 Mb-10.85 Mb) and *Ras association domain-containing protein 10* (11.40-11.41 Mb) were also identified for having over a three-fold male-biased expression pattern. Ras association domain family proteins have been implicated as tumor suppressors [47–49]. Therefore, upregulation of these genes could suppress primordial germ cell proliferation leading to maleness.

Lastly, it is possible that there could be Y-specific genes that were not captured in our study, because the reference genome that the reads were aligned to is a homozygous clonal XX individual.

## Conclusions

Inversions have been well-documented in sex-chromosome evolution and are one possible mechanism for resolving sexually antagonistic selection near the novel sex-determiner through a reduction in recombination [4]. This study revealed an 8.8 Mb block of differentiation between males and females. The variety of evidence presented here is most consistent with the presence of an inversion. The decay of genes and overall level of differentiation indicate that this region has substantially reduced recombination. We have also documented an accumulation of SNPs causing functional alterations within this region, as would be expected for a genomic region suffering both the deleterious effects of Muller’s Ratchet and accumulation of deleterious alleles hitchhiking to fixation with advantageous alleles. The transcriptome data indicates that genes inside the proposed inversion show significant enrichment for female-biased expression. These data suggest that *O. niloticus* has not yet evolved complete dosage compensation. Future functional studies are needed to identify the master sex-determination gene(s) within this region. Further research on cichlid sex determination will help unravel the underlying sex-determination network that underlies the rapid turnover of sex-determination mechanisms within teleosts.

## Methods

### Genomic DNA pools

All animal procedures were conducted in accordance with University of Maryland IACUC Protocol #R-10-73. The fish sequenced are 3rd generation descendants of fish collected from a commercial tilapia farm in Amherst, Massachusetts USA. Individuals from two related lab-raised families were sacrificed and visually inspected for the presence testes or ovaries to determine the sex of each fish. Fish with ambiguous or immature gonads were excluded from the study. DNA was extracted from fin clips using a standard phenol/chloroform protocol. To confirm the family identity we genotyped each individual for two sex-linked microsatellite markers selected from the Broad anchored tilapia assembly on linkage group 1 (MS1045 at 14.32 Mb and MS1141 at 15.53 Mb). 33 males and 20 females from family BYL078 and 25 males and 13 females from family BYL084 were selected for pooling. DNA from each individual was then quantified by Picogreen fluorescence on a BioTek FLx800 spectrophotometer and appropriate dilutions were made to ensure equal representation of each individual in the pooled samples. The pooled male (or female) DNA from each family was sheared to 500 bp using a Covaris shearer and indexed separately during library construction. Paired-end libraries for each family/sex were constructed for Illumina sequencing using the Illumina TruSeq DNA Sample Prep Kit (Illumina, San Diego, CA). The male (or female) libraries from each family were combined and each sex was sequenced in a separate lane on an Illumina HiSeq 2000. The male and female reads were deposited to NCBI with the accession numbers SRR1606298 and SRR1606304, respectively. Only reads passing the Illumina CASAVA filtering were retained.

Read qualities was checked with FASTQC [50]. Alignments to the *O. niloticus* anchored reference assembly [51] were performed with Bowtie 2 [52] using the *--very-sensitive* setting (Additional file 5). The mean alignment rate was 90.12% in males and 90.67% in females (Separate values for each family are given in Additional file 5). Read alignments were filtered for a minimum mapping quality (MAPQ) of 20 before further analysis. Insert sizes were analyzed using Picard tools CollectInsertSizeMetrics package [53]. The aligned mean insert size was 188.76 bp (standard deviation = 44.81 bp) for males and 167.62 bp (standard deviation = 37.29 bp) for females. Variants were called using GATK [54].

### Genomic analysis

Popoolation2 [55] was used to calculate F_ST_ and Fisher’s exact test on allele frequency differences between the male and female pools. Initial F_ST_ results from the individually adapter-indexed families were very similar, so all subsequent analyses were performed on the combined male or female pool, including unassigned reads from the male and female lane which could not be assigned to a particular family.

A custom Perl script, Sex_SNP_finder_now.pl (available at https://github.com/Gammerdinger/sex-SNP-finder), was used to identify SNPs at intermediate frequencies in the male pools and were fixed or nearly fixed in female pools at the same position. Intermediate SNPs were defined as SNPs with a frequency between 0.3 and 0.7 within the male pool. Fixed or nearly fixed sites required a frequency less than or equal to 0.1 or greater than or equal to 0.9 within the female pool. We used a non-overlapping window of 10 kb to determine the density of these SNPs. The non-overlapping window did not include positions with coverage less than 10 reads in both sexes. The Sex_SNP_finder_now.pl script outputs a tab-delimited file with the number of SNPs per window along with an Integrative Genomics Viewer file [56] that lists all SNPs that were fixed or nearly fixed in one designated pool and in intermediate frequency in the other.

We used SnpEff [30] to identify variants predicted to alter gene function. The SnpEff output was filtered to consider only the SNPs found using Sex_SNP_finder_now.pl. SnpSift [31] was used to extract out SNPs with similar effects and impacts. A complete list of genes within the proposed inversion can be found in Additional file 6.

### Transcriptome analysis

Gonads were dissected from individual larvae 28 days post-fertilization. The sex of each larvae was determined by genotyping microsatellite markers highly associated with sex. RNA from approximately 20 male or 20 female larvae was pooled and cDNA libraries were constructed using the Illumina TruSeq DNA Sample Prep Kit. Sequencing of these libraries yielded ~392 million reads for each male and female pool. Reads were aligned to the *O. niloticus* reference sequence with TopHat2 [57]. NCBI RefSeq annotations were used to guide the Cufflinks [58] assembly (-g) and Cuffdiff was used to was used to determine FPKM values for those gene models. The results were subsequently filtered to exclude gene models whose FPKM value was less than 0.05 in both males and females. Additionally, when comparisons between FPKM of the two sexes was carried out, if the FPKM value exceeded 0.05 in one sex and was zero in the other sex, it was considered an undefined bias favoring the sex with expression. Female-biased and male-biased gene models from inside and outside the proposed inversion were counted and statistical significance was looked for using χ^2^ with Yates’ correction on a 2x2 contingency table. These male and female reads from the RNA-Seq experiment were deposited to NCBI with the accession numbers SRR1606274 and SRR1606273, respectively.

## Electronic supplementary material

Additional file 1: **Putative functional mutations outside the proposed inversion.** List of the genes outside the inversion that contain a stop codon or splice site alteration that was in intermediate frequency in males and fixed or nearly fixed in females. There were no start codon alterations meeting these requirements found outside of the proposed inversion. (XLSX 40 KB)

Additional file 2: **Missense mutations from the entire genome.** List of the missense mutations from the entire genome and their locations which were in intermediate frequency in males and fixed or nearly fixed in females. (XLSX 56 KB)

Additional file 3: **Gene models with male-biased expression.** Male-biased gene locations, male and female FPKM values and log_2_ ratio of male to female FPKM. Thick bold lines indicate groups of genes showing two-fold, three-fold or four-fold expression difference. The genes are ordered from highest male-bias to lowest male-bias. *A second gene model of XM_003437627.1 has a female-biased expression. **A second gene model of XM_003442371.1.1 has a female-biased expression. (XLS 45 KB)

Additional file 4: **Gene models with female-biased expression.** Female-biased genes locations, male and female FPKM values and log_2_ ratio of female to male FPKM. Thick bold lines indicate groups of genes showing two-fold, three-fold or four-fold expression difference. The genes are ordered from highest female-bias to lowest female-bias. *Two gene models of XM_003442373.1 have a female biased expression. **A separate gene model of XM_003437627.1 has a high male-biased expression. ***A second gene model of XM_003442371.1.1 has a female-biased expression. (XLS 69 KB)

Additional file 5: **Bowtie2 alignment statistics.** Alignment statistics for each sex within each family along with the alignment statistics for the pooled data after the families and unassigned reads were combined. (DOC 25 KB)

Additional file 6: **List of genes inside proposed inversion.** NCBI accession numbers and gene names for the genes identified within the proposed inverted region. (XLSX 20 KB)
