# Supplementary material for: Structure and decay of a proto-Y region in Tilapia, Oreochromis niloticus
Source: BMC Genomics. 2014 Nov 17;15(1):975. doi: 10.1186/1471-2164-15-975 (PMC4251933; doi:10.1186/1471-2164-15-975)
Supplement: Supplementary file 5 — Additional file 5: Bowtie2 alignment statistics. Alignment statistics for each sex within each family along with the alignment statistics for the pooled data after the families and unassigned reads were combined. (DOC 25 KB) [file 12864_2014_6700_MOESM5_ESM.doc]

**Bowtie2 Alignment Statistics**

**BYL078 Males**

118613332 reads; of these:

118613332 (100.00%) were paired; of these:

79617996 (67.12%) aligned concordantly 0 times

30036951 (25.32%) aligned concordantly exactly 1 time

8958385 (7.55%) aligned concordantly >1 times

----

79617996 pairs aligned concordantly 0 times; of these:

45642099 (57.33%) aligned discordantly 1 time

----

33975897 pairs aligned 0 times concordantly or discordantly; of these:

67951794 mates make up the pairs; of these:

17809331 (26.21%) aligned 0 times

9514821 (14.00%) aligned exactly 1 time

40627642 (59.79%) aligned >1 times

92.49% overall alignment rate

**BYL084 Males**

75692818 reads; of these:

75692818 (100.00%) were paired; of these:

59812679 (79.02%) aligned concordantly 0 times

12186442 (16.10%) aligned concordantly exactly 1 time

3693697 (4.88%) aligned concordantly >1 times

----

59812679 pairs aligned concordantly 0 times; of these:

32435641 (54.23%) aligned discordantly 1 time

----

27377038 pairs aligned 0 times concordantly or discordantly; of these:

54754076 mates make up the pairs; of these:

15935205 (29.10%) aligned 0 times

9706650 (17.73%) aligned exactly 1 time

29112221 (53.17%) aligned >1 times

89.47% overall alignment rate

**BYL078 Females**

140230450 reads; of these:

140230450 (100.00%) were paired; of these:

113114176 (80.66%) aligned concordantly 0 times

20544442 (14.65%) aligned concordantly exactly 1 time

6571832 (4.69%) aligned concordantly >1 times

----

113114176 pairs aligned concordantly 0 times; of these:

68713992 (60.75%) aligned discordantly 1 time

----

44400184 pairs aligned 0 times concordantly or discordantly; of these:

88800368 mates make up the pairs; of these:

20281912 (22.84%) aligned 0 times

10358194 (11.66%) aligned exactly 1 time

58160262 (65.50%) aligned >1 times

92.77% overall alignment rate

**BYL084 Females**

73053493 reads; of these:

73053493 (100.00%) were paired; of these:

63524963 (86.96%) aligned concordantly 0 times

7220466 (9.88%) aligned concordantly exactly 1 time

2308064 (3.16%) aligned concordantly >1 times

----

63524963 pairs aligned concordantly 0 times; of these:

36660770 (57.71%) aligned discordantly 1 time

----

26864193 pairs aligned 0 times concordantly or discordantly; of these:

53728386 mates make up the pairs; of these:

15062835 (28.04%) aligned 0 times

7909966 (14.72%) aligned exactly 1 time

30755585 (57.24%) aligned >1 times

89.69% overall alignment rate

**Combined Males**

201737120 reads; of these:

201737120 (100.00%) were paired; of these:

38855185 (19.26%) aligned concordantly 0 times

123497206 (61.22%) aligned concordantly exactly 1 time

39384729 (19.52%) aligned concordantly >1 times

----

38855185 pairs aligned concordantly 0 times; of these:

4324802 (11.13%) aligned discordantly 1 time

----

34530383 pairs aligned 0 times concordantly or discordantly; of these:

69060766 mates make up the pairs; of these:

39856932 (57.71%) aligned 0 times

14127413 (20.46%) aligned exactly 1 time

15076421 (21.83%) aligned >1 times

90.12% overall alignment rate

**Combined Females**

219436845 reads; of these:

219436845 (100.00%) were paired; of these:

37879573 (17.26%) aligned concordantly 0 times

135977644 (61.97%) aligned concordantly exactly 1 time

45579628 (20.77%) aligned concordantly >1 times

----

37879573 pairs aligned concordantly 0 times; of these:

4923549 (13.00%) aligned discordantly 1 time

----

32956024 pairs aligned 0 times concordantly or discordantly; of these:

65912048 mates make up the pairs; of these:

40931843 (62.10%) aligned 0 times

11732275 (17.80%) aligned exactly 1 time

13247930 (20.10%) aligned >1 times

90.67% overall alignment rate
